# Implications of Hybridization, NUMTs, and Overlooked Diversity for DNA Barcoding of Eurasian Ground Squirrels

**DOI:** 10.1371/journal.pone.0117201

**Published:** 2015-01-24

**Authors:** Oleg A. Ermakov, Evgeniy Simonov, Vadim L. Surin, Sergey V. Titov, Oleg V. Brandler, Natalia V. Ivanova, Alex V. Borisenko

**Affiliations:** 1 Department of Zoology and Ecology, Penza State University, Penza, Russia; 2 Institute of Systematics and Ecology of Animals, Siberian Branch of the Russian Academy of Sciences, Novosibirsk, Russia; 3 Tomsk State University, Tomsk, Russia; 4 Hematological Research Center, Russian Academy of Medical Sciences, Moscow, Russia; 5 Koltzov Institute of Developmental Biology, Russian Academy of Sciences, Moscow, Russia; 6 Biodiversity Institute of Ontario, University of Guelph, Guelph, Canada; The University of Western Australia, AUSTRALIA

## Abstract

The utility of DNA Barcoding for species identification and discovery has catalyzed a concerted effort to build the global reference library; however, many animal groups of economical or conservational importance remain poorly represented. This study aims to contribute DNA barcode records for all ground squirrel species (Xerinae, Sciuridae, Rodentia) inhabiting Eurasia and to test efficiency of this approach for species discrimination. Cytochrome *c* oxidase subunit 1 (COI) gene sequences were obtained for 97 individuals representing 16 ground squirrel species of which 12 were correctly identified. Taxonomic allocation of some specimens within four species was complicated by geographically restricted mtDNA introgression. Exclusion of individuals with introgressed mtDNA allowed reaching a 91.6% identification success rate. Significant COI divergence (3.5–4.4%) was observed within the most widespread ground squirrel species (*Spermophilus erythrogenys*, *S. pygmaeus*, *S. suslicus*, *Urocitellus undulatus*), suggesting the presence of cryptic species. A single putative NUMT (nuclear mitochondrial pseudogene) sequence was recovered during molecular analysis; mitochondrial COI from this sample was amplified following re-extraction of DNA. Our data show high discrimination ability of 100 bp COI fragments for Eurasian ground squirrels (84.3%) with no incorrect assessments, underscoring the potential utility of the existing reference librariy for the development of diagnostic ‘mini-barcodes’.

## Introduction

DNA barcoding [1] has proved to be a useful tool for species identification (e.g. [2–5]) and serving for various needs from forensic analysis (e.g. [6]) to biodiversity surveys (e.g. [7]). The 5-prime ~650 base pair region of the mitochondrial cytochrome oxidase subunit I gene (COI) is the generally accepted standard DNA barcode marker for most species of animals. Some applications, like noninvasive analysis of old samples (e.g. [8]) or the emerging DNA metabarcoding [9], require shorter markers (~50–200 bp). An important challenge to this approach is posed by the introgression of mitochondrial DNA due to hybridization and/or incomplete lineage sorting of mtDNA haplotypes (e.g. [10]). Both of them can lead to the absence of the “barcoding gap” and cause misidentification. An essential prerequisite in the utility of DNA barcoding for practical applications is the creation of a high-quality reference database that is scrutinized for possible analytical and taxonomic errors. Despite a concerted effort to attain broad representation of key taxa in the reference library hosted by the Barcode of Life Data System (BOLD—www.boldsystems.org) many groups of economical or conservational importance remain poorly represented.

Ground squirrels (Marmotini) are a charismatic faunal element of grassland communities across north-temperate biomes of the Holarctic and play an important role in maintaining these open habitats. In Eurasia, this group is represented by 16 species belonging to the genera *Spermophilus* and *Urocitellus* [11]. *Spermophilus* has exclusively Palaearctic distribution, while *Urocitellus* is predominantly Nearctic, with only two species occurring in Easternmost Siberia. The native range of Eurasian ground squirrels spans a vast area from Central Europe and the Middle East to the Chukotka Peninsula [12, 13]. Historically, these animals have been regarded as major agriculture pests [14]. In addition, they were found to be important reservoirs of dangerous natural-focal zoonotic infections, such as plague, rabbit-fever, relapsing fever, Q fever, brucellosis, etc. [15–17]. Triggered by these findings, concerted eradication efforts have been deployed across of Eurasia throughout much of the XX century [18]. Coupled with extensive agricultural transformation of grassland habitats, this has led to significant population decline and range fragmentation in many ground squirrel species (e.g. [19, 20]).

Today, the trend has shifted from extermination to protection, which is manifested by a growing number of ground squirrel conservation and reintroduction programs in parts of Europe (e.g. [21, 22]). Several Eurasian ground squirrels have special global conservation status in the IUCN Red List: three species (*S. musicus*, *S. suslicus* and *S. xanthoprymnus*) are listed as ‘Near Threatened’ and one (*S. citellus*) – as ‘Vulnerable’ [23]. Many Central and East European populations are facing local threats from human environmental impact and are included in national and regional Red Data Lists; some of them are presently believed to be extinct [24]. For instance, the Red-cheeked ground squirrel *(S. erythrogenys*) is now extinct from its type locality (our data). Assessment of conservation status in some ground squirrels is hampered by on-going debates about the taxonomic rank of certain named forms [25, 26]. The recent description of a new species from Turkey *– S. taurensis* [27, 28] suggests that taxonomic knowledge gaps remain even within this relatively well studied group of mammals. The existence of unresolved systematic questions, combined with conservational and epizootological importance of Eurasian ground squirrels calls for continued taxonomic reassessments employing novel methodological approaches and for the development of new diagnostic tools.

This study aims to establish the COI barcode reference library for all ground squirrel species inhabiting Eurasia, to assess its utility for species discrimination, to highlight any previously unrecognised genetic diversity, and to discuss possible implications of mitochondrial introgression.

## Materials and Methods

### Sample collection

**Figure 1:**
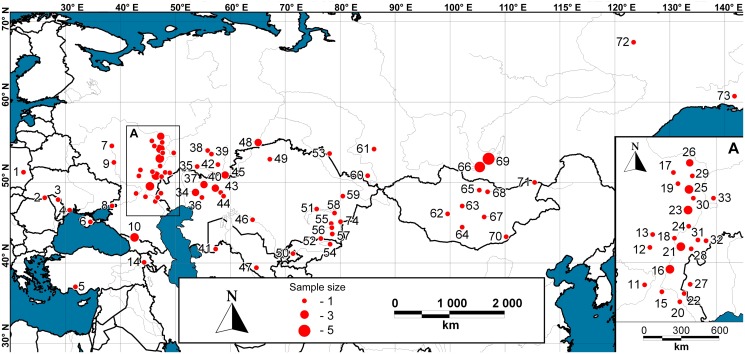
Map of the study area showing collection localities for this study (see S1 Table for locality information).

The studied material represents all 16 presently recognized ground squirrel species from Eurasia (genera *Spermophilus* and *Urocitellus*) and includes all species from the former genus (Fig. 1). Geographic sampling spans 74 different locations. No experiments were conducted with living animals. All ground squirrel and “outgroup” samples came from preserved tissue from vouchered collection specimens deposited in the following institutions: Penza State University (PSPU; 69 samples); Koltzov Institute of Developmental Biology, Russian Academy of Sciences (IDB; 14 samples); Zoological Museum of Moscow State University (ZMMU; 11 samples); Zoological Museum, Institute of Systematics and Ecology of Animals, Siberian Branch, Russian Academy of Sciences (ISEA; five samples); Charles University in Prague (CU; one sample); Zoological Institute, Russian Academy of Sciences (ZISP; one sample). Museum catalogue numbers along with repository and locality data are given in S1 Table. All specimens used in this study were morphologically identified prior to sequencing; taxonomy follows Helgen *et al*. [11].

### DNA isolation, amplification and sequencing

Extraction of total DNA and subsequent analyses was done either at the Biodiversity Institute of Ontario, Guelph, following protocols provided in Ivanova *et al*. [29], or at the Laboratory of Animal Systematics and Molecular Ecology, Penza State University, following the protocols described below. DNA was extracted according to a standard procedure including the treatment with sodium dodecyl sulphate and proteinase K, and subsequent phenol-chloroform extraction [30]. To deal with degraded DNA from old museum samples, we developed two primer pairs specific to ground squirrels (subfamily Marmotinae): Sp-COXD – 5’-GAT GAT TCT TCT CAA CTA ATC-3’ and SpCOXRr – 5’-CAT GGG CRA GAT TTC CAG CTA-3’; SpCOXDd – 5’-CTT CTA TRG TTG AAG CAG GTG C-3’ and SpCOXR – 5’-TGA GAA ATT ATA CCA AAT CCT G-3’. Each PCR reaction contained 50 mM Tris–HCl (pH 8.9), 20 mM ammonium sulphate, 20 μM EDTA, 150 µg/ml bovine serum albumin, dNTPs (200 µM of each), 2 mM MgCl_2_, 15 pmol of each primer, 2 units of *Taq* polymerase and 0.1 to 0.2 µg DNA in a final volume of 25 µl. The reaction conditions were 94°C for 1 min; 62°C for 1 min; and 72°C for 1 min (30 cycles). PCR products were analysed using electrophoresis in 6% PAAG with subsequent staining with ethidium bromide and visualization in the UV light. Sequencing was done on an ABI 3500 automated capillary sequencer (Applied Biosystems) with the ABI Prism Big Dye Terminator Cycle Sequencing Ready Reaction Kit 3.1 using the same primers. Sequences were aligned manually and checked for unexpected stop codons using BioEdit 7.0 [31].

### Data analysis

To complement our analysis, additional sequences of New World ground squirrels and other selected members of the family Sciuridae were obtained from the BOLD project “Mammals of Canada” (ABMC) and from GenBank (S1 Table). Although DNA barcoding is not a phylogenetic approach, we used MetaPIGA2 [32] to infer the gene tree using Maximum Likelihood (ML) that was compared against the branching pattern inferred from the ‘conventional’ Neighbour-Joining (NJ) method. Before running ML analysis, the dataset was tested for redundancy and transition saturation using the same program. The default substitution model used by BOLD (www.boldsystems.org) is K2P model [33]; however the use of this model in DNA-barcoding has been criticized (i.e. [34, 35]). Thus, we determined the best-fitting models of nucleotide substitution for our data using jModelTest 2.1.1 [36] with Akaike Information Criterion (AIC). We used a variable number of bootstrap replicates, stopping the iterations when the mean relative error among 10 consecutive consensus trees stayed below 5% (minimum 100, maximum 10 000). Tree topologies resulting from ML analyses were visualised and edited using FigTree v.1.4 [37].

Intra- and interspecies genetic distances (*p*-distances) and their standard errors based on 1000 bootstrap replications were calculated using MEGA 5.1 [38]. The number of haplotypes, haplotype diversity and nucleotide diversity per site for each species were computed with the help of DnaSP v. 5.10.1 software [39]. To test barcoding efficiency in our dataset, we used the DNA barcoding package Spider v. 1.1–5 [40] for R [41]. Two methods which mimic the *p*-distance-based “species identification” algorithm used by BOLD were applied: the “threshID” function in Spider and the “best close match” criterion of Meier *et al*. [42]. Two approaches were used in threshold selection: the first one included optimisation procedure which minimises false-positive and false-negative errors for a range of threshold values (0.1–4% in 0.1% increments); the second approach is the experimental method implemented in Spider (“localMinima” function) which produces a density object from the distance matrix and determines where a dip in the density of genetic distances indicates the transition between intra- and inter-specific distances. Species represented by only one individual (singletons) were excluded from these analyses. Additionally, we checked the dataset for the presence of the “barcoding gap” [43] by calculating the furthest intraspecific distance and the closest non-conspecific distance for each individual in the dataset [44] and by producing its graphical representation.

In addition, we applied two recently developed methods for automated species delineation to explore possible cryptic diversity in Eurasian ground squirrels. Bayesian implementation of the Poisson tree processes (PTP) model [45] was tested to delimit species on the ML tree generated by MetaPIGA2 (without outgroups), using the bPTP server (http://species.h-its.org) with the following settings: 200 000 MCMC generations; thinning interval of 100 and first 15% was discarded as burn-in. Analysis were run three times with different random seed to ensure consistency of results between runs; a convergence within each run was assessed by examining of a likelihood trace plot. The second approach used was the refined single linkage (RESL) analysis, introduced by Ratnasingham and Hebert in 2013 [46] along with the Barcode Index Number (BIN) system. RESL is a multi-step process serving to assign DNA barcode sequences to operational taxonomic units (OTUs). Then, each OTU is assigned to a uniform resource identifier within the BIN system [46]. These steps were run on BOLD (http://www.boldsystems.org) as part of its operational routine and include all COI records stored in its reference library.

In view of the rapid advent of DNA metabarcoding studies, we examined our dataset for the regions potentially useful for generation of mini-barcodes (50–100 bp long) via the slideBoxplots function implemented in Spider. The distribution of pairwise genetic distances of each window was calculated and plotted for 50-bp and 100-bp width windows using a 3-bp (codon) interval. At the next step, a number of 50- and 100-bp windows with highest divergence were chosen for threshold value optimization and tested for barcoding efficiency as described above.

The data used in this study (sequences, trace files, and associated detailed specimen information) are available online at http://www.boldsystems.org in the published BOLD project “Ground Squirrels of the Palaearctic” (ABGPA). The full dataset containing these records and other published records used for comparison is available as the BOLD dataset “Ground Squirrels of the Palaearctic – Comparative Dataset” (DS-GSPA), DOI: dx.doi.org/10.5883/DS-GSPA. Original sequence data were also deposited in NCBI GenBank, accessions KM537885 – KM537985 (S1 Table).

## Results

**Figure 2:**
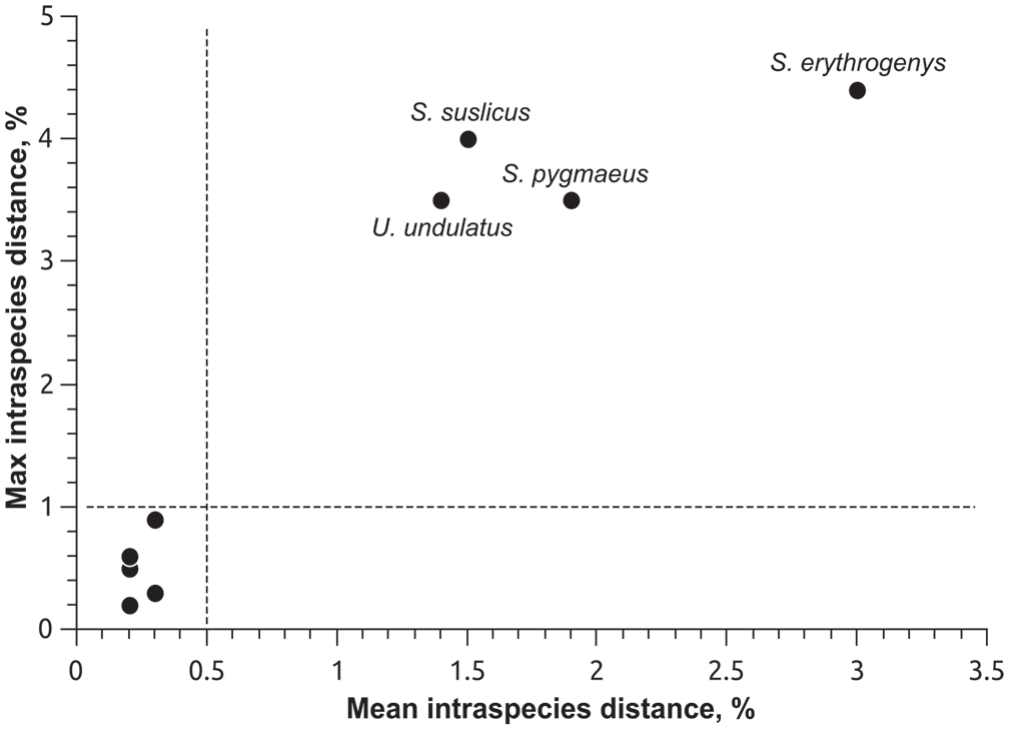
Plot of maximum intraspecies distances against mean intraspecies distances of Eurasian ground squirrels (excluding singletons). Species names are given for putative species complexes indicated by DNA barcoding.

COI barcode sequences (657 bp long) were obtained for 97 individuals of 16 ground squirrel species from 74 locations (Fig. 1). Thus, all ground squirrel species known from Eurasia were covered for the first time. The alignment contained 188 variable positions, of which 166 were parsimony-informative. The mean transition/transversion ratio (over all sequence pairs) was 5.741, and the mean base composition was A: 26.4, C: 23.6, G: 16.2 and T: 33.8%. The mean *p*-distances between species within genera *Spermophilus* and *Urocitellus* were 6.9 and 11.3%, respectively. The mean distance to the nearest neighbour species within all Eurasian ground squirrels ranged from 0.5% (*S. brevicauda* and *S. major*) to 7.9% (*S. xanthoprymnus* and *S. taurensis*), with a mean of 4.4% (Table 1). Maximum intraspecific distances were observed within species with the widest distribution ranges: *S. erythrogenys* (4.4%), *S. suslicus* (4.0%), *S. pygmaeus* (3.5%), and *U. undulatus* (3.5%) (Fig. 2, Table 1).

**Table 1:** Intraspecific genetic variation of Eurasian Ground Squirrels.

**Species name**	**n/N**	***h* ± SD**	***π* ± SD**	**Mean intra-sp ± SE**	**Max intra-sp**	**Nearest species**	**Distance to NS ± SE**
Spermophilus alashanicus	2/2	1.00 ± 0.50	0.002 ± 0.001	0.002 ± 0.002	0.002	S. citellus	0.073 ± 0.010
Spermophilus brevicauda	4/3	0.83 ± 0.22	0.002 ± 0.001	0.002 ± 0.001	0.005	S. major	0.005 ± 0.002
Spermophilus citellus	1/1	-	-	-	-	S. alashanicus	0.073 ± 0.010
Spermophilus dauricus	2/2	1.00 ± 0.50	0.003 ± 0.002	0.003 ± 0.002	0.003	S. alashanicus	0.074 ± 0.010
Spermophilus erythrogenys	4/4	1.00 ± 0.18	0.030 ± 0.009	0.030 ± 0.005	0.044	S. major	0.024 ± 0.004
Spermophilus fulvus	10/5	0.67 ± 0.16	0.003 ± 0.001	0.003 ± 0.001	0.009	S. major	0.032 ± 0.006
Spermophilus major	10/4	0.53 ± 0.18	0.002 ± 0.001	0.002 ± 0.001	0.006	S. brevicauda	0.005 ± 0.002
Spermophilus pallidicauda	2/2	1.00 ± 0.50	0.002 ± 0.001	0.002 ± 0.001	0.002	S. ralli	0.030 ± 0.007
Spermophilus pygmaeus	19/12	0.95 ± 0.03	0.018 ± 0.001	0.019 ± 0.003	0.035	S. pallidicauda	0.071 ± 0.009
Spermophilus ralli	1/1	-	-	-	-	S. major	0.017 ± 0.005
Spermophilus relictus	1/1	-	-	-	-	S. ralli	0.029 ± 0.006
Spermophilus suslicus	15/13	0.97 ± 0.04	0.015 ± 0.004	0.015 ± 0.002	0.040	S. pallidicauda	0.077 ± 0.010
Spermophilus taurensis	1/1	-	-	-	-	S. citellus	0.044 ± 0.008
Spermophilus xanthoprymnus	1/1	-	-	-	-	S. taurensis	0.079 ± 0.011
Urocitellus undulatus	13/6	0.77 ± 0.10	0.014 ± 0.004	0.014 ± 0.003	0.035	U. columbianus	0.055 ± 0.008
Urocitellus parryii	2/2	1.00 ± 0.50	0.002 ± 0.001	0.002 ± 0.001	0.002	U. richardsonii	0.024 ± 0.006

n/N – sample size/number of haplotypes; *h* ± SD – haplotype diversity ± standard deviation; *π* ± SD—nucleotide diversity (per site) ± standard deviation; Mean intra-sp ± SE – mean intraspecies p-distance ± standard error; Max intra-sp – maximum intraspecies p-distance; Distance to NS ± SE – *p*-distance to a nearest species ± standard error (based on 1000 bootstrap replications).

**Figure 3:**
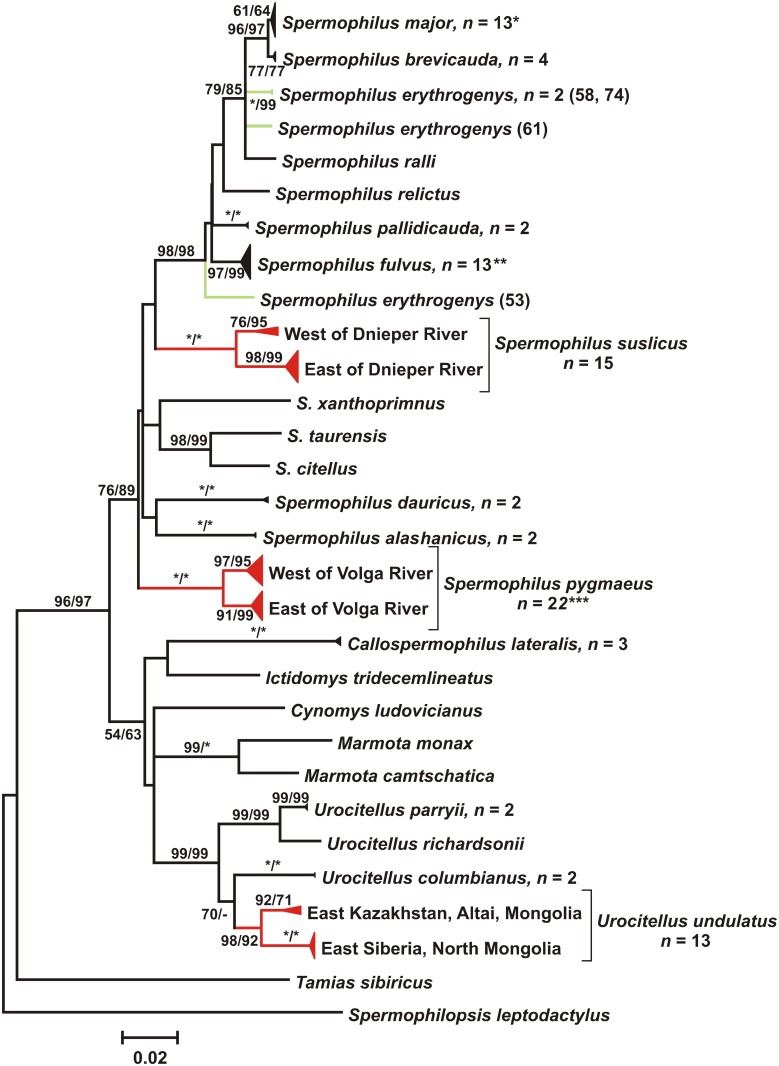
ML tree (HKY+I+G model) for the 657 bp fragment of COI of all Eurasian ground squirrel species. Bootstrap values above 50 are indicated; asterisks represent bootstrap values of 100. The nodes with multiple specimens were collapsed to a triangle, with the horizontal depth indicating the level of intraspecific divergence. Numbers next to each species name indicate the sample size (not indicated when n = 1). * individuals with introgressed mtDNA are not included into this analysis.

According to jModelTest 2.1.1 AIC, the best model for our COI dataset was HKY+I+G (Fig. 3). While recognizing the limitations of a single-gene approach and refraining from inferring phylogenetic conclusions, we note that the obtained tree is in agreement with current views on the taxonomy of ground squirrels and corroborate the findings obtained using another mitochondrial marker – cytochrome *b* [11, 25]. The monophyly of *Spermophilus* and *Urocitellus* was supported by high bootstrap values (76–99%; Fig. 3). Within *Spermophilus*, the grouping of species from the subgenus *Colobotis* also had high bootstrap support (98%). This grouping also included *S. relictus* and *S. ralli* which were previously assigned to the subgenus *Citellus* [47] or conf. *Urocitellus* [48]. On the other hand, the monophyly of the subgenus *Citellus* was not supported.

### mtDNA introgression in Spermophilus

In four ground squirrel species from the Volga region (*S. fulvus*, *S. major*, *S. pygmaeus*, and *S. erythrogenys*) several putative hybridization events were detected (Table 2). In particular, 20% and 13.3% of examined specimens of *S. major* had COI haplotypes of *S. pygmaeus* and *S. fulvus* respectively. In *S. fulvus*, 16.6% of individuals had haplotypes of *S. major*; 5% of *S. pygmaeus* had *S. fulvus* haplotypes; and 20% of *S. erythrogenys* had *S. major* haplotypes.

**Table 2:** Frequency of the introgressed mtDNA haplotypes in four ground squirrel species as revealed by DNA barcoding.

Species	mtDNA haplotypes		
	*S. major*	*S. fulvus*	*S. pygmaeus*
*Spermophilus major*	10	2	3
*Spermophilus fulvus*	2	10	0
*Spermophilus pygmaeus*	0	1	19
*Spermophilus erythrogenys*	1	0	0

### Identification success

The test of barcoding efficiency was performed on the dataset with excluded singletons and individuals harbouring introgressed mtDNA. The “species identification” method (“threshID” in Spider) with a default threshold of 1% allowed the correct identification of 62 individuals, while 14 were ambiguous and seven individuals had no matches (74.7% success rate). The “best close match” approach performed better: it correctly identified 76 individuals (91.6%), while seven ranked as having “no matches”. Optimisation of the threshold value via minimization of false-positive and false-negative errors and using the “localMinima” function showed identical results with an optimal threshold value of 1.7%. Application of the optimal threshold value notably improved identification success with 67 correct, 14 ambiguous and two “no matches” identifications using “threshID” (80.7%) and 81 correct and two “no matches” via “best close match” (97.6%).

**Figure 4:**
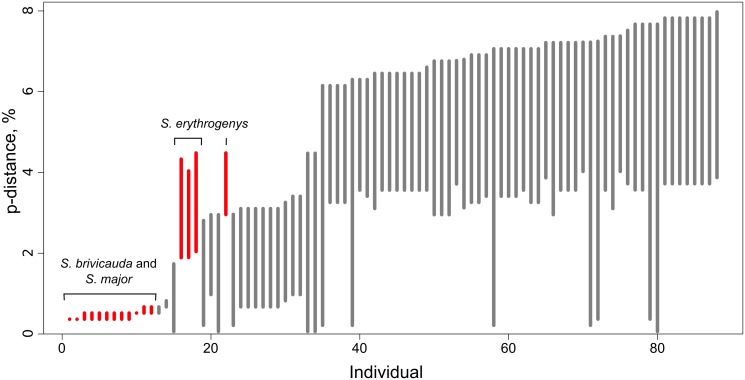
Line-plot of the barcode gap for the Eurasian ground squirrels as generated by Spider [40]. For each individual in the dataset, the grey lines represent the furthest intraspecific distance (bottom of line value), and the closest interspecific distance (top of line value). The red lines show where this relationship is reversed, and the closest non-conspecific is actually closer to the query than its nearest conspecific (i.e. no barcoding gap). Individuals with introgressed mtDNA are not included.

The ‘barcode gap’ between species was present in most cases, *i.e.*, maximum intraspecific distances were smaller than minimum interspecific distances for 72 out of 88 individuals in the dataset, including singletons (Fig. 4). There was no ‘barcode gap’ between *S. major* and *S. brevicauda* (due to small interspecies distances) and for *S. erythrogenys* (due to high intraspecies distances).

### Automatic species delineation

**Figure 5:**
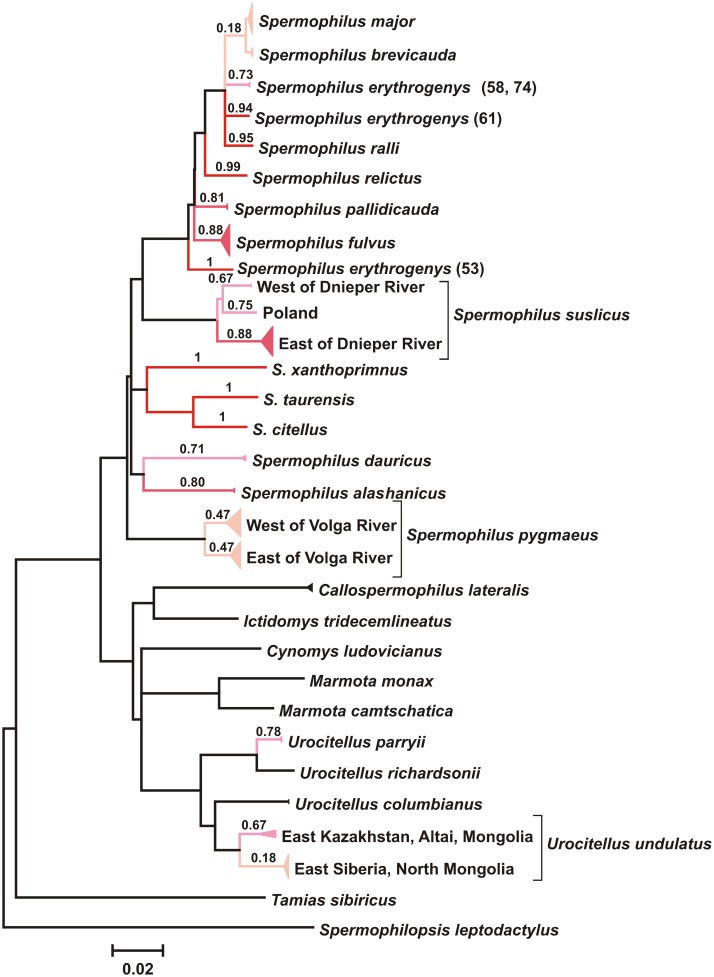
Species delineation of Eurasian ground squirrels by Poisson tree processes (PTP) on the ML tree (HKY+I+G model; 657 bp of COI). Bayesian support values for delimited species are indicated;intensity of red color reflects the strength of support. The nodes with multiple specimens were collapsed to a triangle, with the horizontal depth indicating the level of intraspecific divergence. Individuals with introgressed mtDNAare not included into the tree.

**Figure 6:**
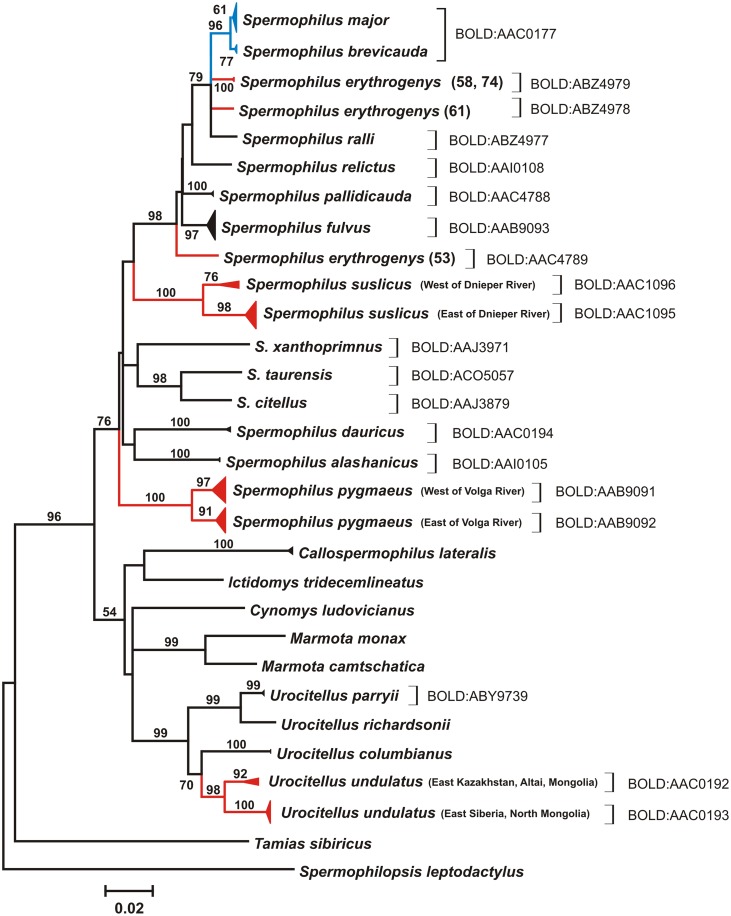
Species delineation of Eurasian ground squirrels by refined single linkage (RESL) analysis showed on the gene tree. Square brackets indicate putative species recognized by RESL. Barcode Index Numbers (BIN) assigned to each putative species are given as BOLD:XXXXXXX. Species split by RESL are red colored, while merged species are blue colored. ML tree (HKY+I+G model) of 657 bp COI fragment. Bootstrap values above 50 are indicated. The nodes with multiple specimens were collapsed to a triangle, with the horizontal depth indicating the level of intraspecific divergence. Individuals with introgressed mtDNA are not included into the tree.

According to bPTP, the estimated number of species was between 17 and 60, with a mean of 36; however, the best maximum likelihood solution provided a more reliable estimate with 21 species identified (Fig. 5). The RESL algorithm implemented in BOLD identified 20 BIN clusters (Fig. 6), while the number of currently recognized ground squirrel taxa in Eurasia is 16. As expected, both approaches split species with observed high intraspecific distances (*S. erythrogenys*, *S. suslicus*, *S. pygmaeus*, and *U. undulatus*) and merged *S. major* and *S. brevicauda*.

### Sliding windows analysis

The analysis using 100 bp and 50 bp sliding windows detected two regions within the COI barcode sequence, potentially useful for the development of diagnostic ‘mini-barcodes’. Results of threshold optimisation and estimations of identification success for the four best 100 bp and 50 bp windows are given in Table 3. The best identification success (84.3%) was with the 100 bp fragment that started from position 46 in the alignment.

**Table 3:** Identification success for the best 100 bp and 50 bp mini-barcodes identified by sliding windows analysis in Spider.

Position*	Width, bp	Optimal threshold, %	Best Close Match				Success rate, %
			ambiguous	correct	incorrect	no id	
40	50	2.1	28	52	1	2	62.7
379	50	1.0	13	61	2	7	73.5
46	100	0.9	0	70	0	13	84.3
322	100	1.1	15	66	1	1	79.5

* position of the first nucleotide of the given window in the alignment.

### Detection of NUMTs

We obtained one putative NUMT (nuclear mitochondrial pseudogene) sequence when analyzing the COI fragment from a fresh ungual phalanx taken from a roadkill *U. undulatus* (KP098531). The NUMT sequence had an unusually high number of nucleotide substitutions (8.8%) and codon deletion (positions 274–276). Furthermore, eight out of 13 CpG-dinucleotides (methylation sites) had mutations in the NUMT sequence, while the same dinucleotides in *U. undulatus* COI sequences carried only three mutations.

## Discussion

### Species delineation and introgression of mtDNA

COI barcode sequences allowed the correct identification of 12 out of 16 Eurasian ground squirrel species; the identification of four species (*S. fulvus*, *S. major*, *S. pygmaeus*, and *S. erythrogenys*) was complicated by mtDNA introgression. When individuals with introgressed mtDNA were removed, the best method for species identification was Meier’s “best close match” criterion that had a 91.6% success rate at a standard threshold of 1%. The procedure of threshold optimization proved to be useful and increased identification success by 6% in both methods. Notably, we found no differences in the performance of different substitution models or their advantage over *p*-distance in species delineation.

Interspecific divergence values observed among Eurasian ground squirrels (6.9% in *Spermophilus* and 11.3% in *Urocitellus*) are congruent with other COI surveys in vertebrates [4, 49–52]. The ‘barcode gaps’ were present in most cases, except *S. brevicauda*, *S. major* and *S. erythrogenys*. In the case of *S. brevicauda* and *S. major*, it was caused by shallow interspecific divergence, while *S. erythrogenys* turned out to be polyphyletic, comprised of genetically distant forms (see discussion below). This was reflected in the results of two automatic (OTU-based) species delineation methods applied to our dataset. Both PTP and RESL analyses merged *S. brevicauda* and *S. major* into single species, while *S. erythrogenys* was split to three species. The performance of these species delimitation methods was very similar, distinguishing more putative species (PTP – 21, RESL – 20) than traditionally recognized through morphological taxonomy (16). In addition to *S. erythrogenys*, these analyses also split *S. suslicus*, *S. pygmaeus*, and *U. undulatus*, in concordance with recent findings on morphological, karyotypic and genetic intraspecies variability of these species (see discussion below).

Barcode-based species recognition was especially hindered in *S. major*, where 33.3% of individuals had COI haplotypes from other species. These findings are not surprising, because the hybridization and mtDNA introgression between these ground squirrel species has been extensively documented [53–58]. Studies of the variability in the D-loop region within a broader sample has revealed 36.7% (52 out of 137 individuals) of *S. major* to possess haplotypes typical for *S. fulvus* and *S. pygmaeus* [59]. The same study found no introgressed haplotypes among 119 individuals of the remainder three species, suggesting predominant participation of *S. major* in backcrosses. Thus, in areas of introgression the identification of these species using mtDNA is complicated and the application of COI alone can lead to misclassification.

The small genetic distance (0.5%) and the absence of barcoding gap between two morphologically distinct species – *S. brevicauda* and *S. major* can be explained by ancient hybridization and introgression of mtDNA. We found prominent nuclear – mitochondrial DNA incongruence by analysing three nuclear loci (gene *p*53 intron 6, gene *bcr* intron 13, and HoxB gene intron 5 – 1636 bp in total; unpublished data). This analysis suggests that *S. brevicauda* and *S. major* are not sister taxa as could be inferred from mtDNA. The most plausible explanation of this pattern is total replacement of native *S. major* mtDNA in the course of past introgression with *S. brevicauda*, followed by divergence. A similar pattern has been recently described in brown and polar bears [60], long-tailed and Menzbier’s marmots [61], serotines [62], and mouse-eared bats, Myotis [63] where ancient hybridization has been inferred from the discordances in mitochondrial and nuclear genome differences.

Introgression events are known to distort the congruence between “species” and “gene” trees [64] and have been identified as a challenge for DNA barcoding. The inability of COI-based barcodes to correctly distinguish between species due to mtDNA introgression was reported in a number of animal taxa [10, 65, 66]; on the other hand, it has been argued that in some of these situations DNA barcodes may still provide adequate resolution for practical identification purposes, provided that the reference library is well populated [52].

Some researchers proposed to use additional nuclear loci in combination with COI. For instance, Raupach *et al*. [66] argued that a combination of COI and nuclear ribosomal expansion segments is an efficient tool for identification of carabid (Coleoptera) species. A number of studies utilized the interphotoreceptor retinoid-binding protein for species delimitation of carnivores and rodent species [8, 67–69]. A similar approach could be reliable for delineation of *Spermophilus* species showing mtDNA introgression, since the frequency of “alien” alleles at nuclear loci in these species is considerably lower [57]. Furthermore, the geographical origin of samples should be taken into account when possible. All *S. major* specimens with introgressed haplotypes originated from an area along the Volga River, approximately 250 km wide. Here, more than 60% *S. major* individuals (n = 96) carry mtDNA from other ground squirrel species [59].

### Intraspecific genetic diversity and divergence

Deep COI barcode divergence was observed within the most widespread ground squirrel species (Fig. 2) and was highlighted in the results of two OTU-based species delineation approaches used in the study (Figs. 5, 6). The range of *S. suslicus* spans 1700 km west to east and is divided by several large rivers [13]. Three to five subspecies have been previously recognized in this species [11, 47, 70]. Furthermore, it is the only species of *Spermophilus* possessing two chromosome races divided by Dnieper River: western (*2n = 36*, *NF = 72*) and eastern (*2n = 34*, *NF = 68*) [71, 72]. Zagorodnuk and Fedorchenko [73] and Korablev [74] proposed that these two races may constitute two different species. Our analysis has revealed a significant level of divergence (up to 4%) between them, which is concordant with an earlier study of D-loop variability that detected 8% genetic distance between these populations [75]. Notably, the PTP analysis split this taxon into three putative species, distinguishing the westernmost population (from Poland) as a separate species (Fig. 5), while RESL discriminated only two taxa divided by Dnieper River (Fig. 6). These results underscore the need for further taxonomic revision of *S. suslicus* s.l., with possible recognition of *S. suslicus* s. str. in the east and *S. odessanus* Nordman, 1840 in the west as distinct species.

Another widespread species showing the east-west split is *S. pygmaeus*. COI barcodes show genetic divergence of up to 3.5% between populations from the west and east banks of the Volga River. Furthermore, both PTP and RESL analyses distinguished them as putative species (Figs. 5, 6). These findings support our previous observations based on the variability of the D-loop region of mtDNA which revealed a 7% genetic distance between them [56] and suggested the subspecies status of the Caucasian mountain ground squirrel (*S. p. musicus*) [76], previously treated as a distinct species. Deep divergence between these two groups is congruent with paleontological data that shows *S. pygmaeus* to have a substantial level of morphological variability as early as Upper Pleistocene [47].

*S. erythrogenys* sensu lato also has a wide range and consists of a number of morphologically different forms with unclear systematics and taxonomy. Gromov *et al*. [47] considered *S. erythrogenys* to be a polymorphic species with a number of subspecies, but Ognev [13] and Sludskiy *et al*. [77] distinguished several independent species. COI barcodes show a mean intraspecific *p*-distance of 3.0% (max 4.4%), with splits into several clusters distinguished by both automatic species delimitation approaches used (Figs. 5, 6). These findings support the opinion that *S. erythrogenys* s. l. represents a species complex; however, the relationships between COI haplogroups within it are more complex than the simple east-west splits associated with prominent dispersal barriers. At least three taxonomic units of putative species rank could be identified: (1) *erythrogenys* (right bank of the Irtysh River) (№ 53, Fig. 3); (2) previously unknown form from the right bank of Ob’ River (Kuznetsk Depression) (№ 61, Fig. 3); (3) *carruthersi* (Zaysan Depression, Dzungarian Alatau) (№ 58, 74, Fig. 3).

*U. undulatus* is the most widespread species of the genus *Urocitellus* inhabiting Eurasia. Its ancestors migrated from North America to north Asia in Upper Pliocene – Lower Pleistocene [78]. Six subspecies of *U. undulatus* are currently recognized [11, 47]. Craniometric data [79] and RAPD-PCR [80] suggest that western and eastern subspecies comprise two groupings of species rank: *S. (u.) undulatus* in the east and *S. (u.) eversmanni* in the west, with the border between lying in Lake Baikal area and northern Mongolia [81, 82]. Our DNA barcode data show a 3.5% genetic distance between these groupings, and both PTP and RESL species delineation approaches identified two putative species within this taxon (Figs. 5, 6).

### NUMTs

Nuclear copies of mitochondrial DNA (NUMTs) are considered a challenge in using mitochondrial DNA for species diagnosis in DNA barcoding [83–86]. NUMTs can be inadvertently amplified while targeting mitochondrial loci, particularly with broad-range primers, and may bias the final dataset. They have been found in over 64 species [85]. Some have suggested that NUMTs make the barcoding approach unreliable, at least in primates [87]. The low frequency (0.9%) of paralogous nuclear COI sequences in our study suggests that this complication may have been over-stated and does not pose problems with ground squirrels. In our dataset the observed case of NUMT detection was overcome by re-extraction of DNA followed by amplification and sequencing under usual conditions.

### Mini-barcodes

Mini-barcodes are small (ca. 100–400 bp) COI gene fragments that have been proposed for use in cases when full-length barcodes (650 bp) could not be retrieved from archived specimens and processed biological material [88]. Today, the increased use of next-generation sequencing platforms broadened the scope and utility of mini-barcodes for species identification proposes. It has been used for the analysis of soil DNA from past and present ecosystems [89] and for diet assessment (reviewed in [90]). Although many of these studies have used alternative DNA markers (e.g. 12S, 16S, ITS1, cyt *b*), there is a growing body of literature using COI sequencing in NGS approaches (e.g., [5]). Our analysis demonstrated high discrimination ability of 100 bp mini-barcodes for Eurasian ground squirrel species (84.3%) with no incorrect assessments. It underscores the utility of existing COI barcode libraries (www.boldsystems.org) for the development of mini-barcodes and their use in species identification.

### Conclusion

Overall, the results of this study provide evidence for the ability of DNA-barcodes to identify most species of Eurasian ground squirrels. Limitations of this approach involve several cases of geographically restricted mtDNA introgression and one case of species polyphyly (in *S. erythrogenys*). The incorporation of nuclear markers and a more fine-grained geographic representation of samples in the reference library should improve identification of *Spermophilus* species engaged in hybridisation and mtDNA introgression. The existence of several genetically divergent haplogroups within several species with wide distribution ranges calls for their in-depth taxonomic reassessment.

## Supporting Information

S1 Table Information on collecting localities, GenBank and BOLD accessions for all specimens used in this study (XLS). (XLS)
