# Correction: Implications of Hybridization, NUMTs, and Overlooked Diversity for DNA Barcoding of Eurasian Ground Squirrels

**DOI:** 10.1371/journal.pone.0120631

**Published:** 2015-04-02

**Authors:** The PLOS ONE Staff

The Funding statement was erroneously left out of this publication. The publisher apologizes for this error. The Funding statement should read:

"This research was supported by the Russian Foundation for Basic Research (RFBR; grant 14-04-00301) to SVT. Funding for DNA barcoding analysis at the Biodiversity Institute of Ontario was provided by grants from the Natural Sciences and Engineering Research Council of Canada (NSERC), Genome Canada through the Ontario Genomics Institute (2008-OGI-ICI-03), the Ontario Ministry of Research and Innovation Global Leadership Program and the Gordon and Betty Moore Foundation to Paul Hebert. Additional funding was provided by the Ministry of Education and Science of the Russian Federation to the Penza State University to conduct scientific activity in 2014–2016."

The Competing Interests statement was also erroneously left out of this publication. The publisher apologizes for this error. The Competing Interests statement should read: The authors have declared that no competing interests exist.
